# GM-CSF-activated STAT5A regulates macrophage functions and inflammation in atherosclerosis

**DOI:** 10.3389/fimmu.2023.1165306

**Published:** 2023-10-18

**Authors:** Jan Nagenborg, Han Jin, Adele V. Ruder, Lieve Temmerman, Barend Mees, Casper Schalkwijk, Daniel Müller-Klieser, Thorsten Berg, Pieter Goossens, Marjo M. P. C. Donners, Erik A. L. Biessen

**Affiliations:** ^1^Department of Pathology, Cardiovascular Research Institute Maastricht (CARIM), Maastricht University Medical Center (UMC), Maastricht, Netherlands; ^2^Department of Vascular Surgery, Maastricht University Medical Center+ (MUMC+), Maastricht, Netherlands; ^3^Cardiovascular Research Institute Maastricht (CARIM), University Maastricht, Maastricht, Netherlands; ^4^Institute for Organic Chemistry, Faculty of Chemistry and Mineralogy, Leipzig, Germany; ^5^Institute for Molecular Cardiovascular Research, Rheinisch-Westfälische Technische Hochschule (RWTH) Aachen University, Aachen, Germany

**Keywords:** STAT5A, STAT5B, GM-CSF, macrophage, atherosclerosis, inflammation

## Abstract

**Introduction:**

Inhibition of STAT5 was recently reported to reduce murine atherosclerosis. However, the role of STAT5 isoforms, and more in particular STAT5A in macrophages in the context of human atherosclerosis remains unknown.

**Methods and results:**

Here, we demonstrate reciprocal expression regulation of STAT5A and STAT5B in human atherosclerotic lesions. The former was highly upregulated in ruptured over stable plaque and correlated with macrophage presence, a finding that was corroborated by the high chromosomal accessibility of STAT5A but not B gene in plaque macrophages. Phosphorylated STAT5 correlated with macrophages confirming its activation status. As macrophage STAT5 is activated by GM-CSF, we studied the effects of its silencing in GM-CSF differentiated human macrophages. STAT5A knockdown blunted the immune response, phagocytosis, cholesterol metabolism, and augmented apoptosis terms on transcriptional levels. These changes could partially be confirmed at functional level, with significant increases in apoptosis and decreases in lipid uptake and IL-6, IL-8, and TNFa cytokine secretion after STAT5A knockdown. Finally, inhibition of general and isoform A specific STAT5 significantly reduced the secretion of TNFa, IL-8 and IL-10 in ex vivo tissue slices of advanced human atherosclerotic plaques.

**Discussion:**

In summary, we identify STAT5A as an important determinant of macrophage functions and inflammation in the context of atherosclerosis and show its promise as therapeutic target in human atherosclerotic plaque inflammation.

## 1 Introduction

Signal transducer and activator of transcription (STAT)5 is a member of the STAT family of transcription factors. STAT5 is essential for differentiation and survival of myeloid cells (1, 2) and has been shown to mediate mammalian target of rapamycin regulated development of monocytes (3). Inhibition of STAT5 activity was recently shown to attenuate atherogenesis by suppressing oxidized low-density lipoprotein (oxLDL)-induced inflammation in murine macrophages (4). The STAT5 subfamily comprises two highly homologous forms, encoded by the STAT5A and STAT5B gene isoforms; these isoforms are located in opposite direction at only 11kb distance, on chromosome 17 (5). Both members were shown to transduce signaling pathways induced by specific cytokines and growth factors, such as interleukin (IL-) 3, IL-5, and granulocyte-macrophage colony-stimulating factor (GM-CSF) (6). Although both forms share 94% of their sequences, targeted gene disruptions in mice yield distinctive phenotypes (5); indeed, several non-redundant functions have been reported (7) with critical roles in proliferation, apoptosis, and cell survival (8). In macrophages, STAT5 is activated by GM-CSF via the Janus kinase 2 (JAK2) (9). Next to M-CSF, GM-CSF is the most important mitogen for macrophage differentiation. Unlike M-CSF, it is mainly expressed in inflammatory conditions (except for lung) to induce a pro-inflammatory macrophage phenotype (10). In line with previous studies, STAT5A deficiency led to defective proliferation and transcriptional reprogramming in GM-CSF exposed murine macrophages (11). While the role in proliferation, survival and differentiation in various cell types is well documented, it is still unknown how STAT5 and in particular its isoforms affect macrophage function and their inflammatory phenotype in humans, especially in a setting of chronic inflammation such as in atherosclerosis.

In this study, we have investigated the regulation of STAT5 and its isoforms in human macrophages and atherosclerotic lesions and their relevance for macrophage transcriptional makeup, activation, and functions relevant to the pathogenesis of atherosclerosis.

## 2 Materials and methods

### 2.1 Human plaque sample collection and morphology

Atherosclerotic plaque samples obtained during carotid endarterectomy [CEA, stratified into non-intraplaque hemorrhage (non-IPH) and IPH groups, IPH being a hallmark of plaque stability and event risk (12, 13)] from 24 symptomatic patients were collected as described previously (Jin et al., 2021 (14)). Tissue collection was in line with the Dutch Code for Proper Secondary Use of Human Tissue (http://www.fmwv.nl) and the local Medical Ethical Committee (protocol number 16-4-181). The endarterectomy specimens were cut into parallel, transverse segments of 5 mm thickness. Each alternating segment was snap-frozen in liquid nitrogen and stored at -80°C, with their flanking segments fixed for 24 hours in formalin, decalcified for 4 hours before processing and embedding in paraffin for histological evaluation.

Only CEA specimens that harbored stable and unstable plaque segments as judged from macroscopic examination (smooth vs rough, thrombotic surface) were included for omics and histological analysis. Plaque tissue segments, based on the absence/presence of IPH as assessed by computer-aided quantitative measurement of extravascular erythrocyte deposits in the Hematoxylin-Eosin (H&E)-stained section, were flanking the omics section. Samples that did not meet the RNA quality criterion (RIN value > 6.0 and A260/280 ratio higher than 1.8) or failed due to technical limitations were excluded. A total of 42 samples (16 IPH^−^ and 26 IPH^+^) were successfully profiled (for details see Jin et al., 2021 (14)).

### 2.2 Transcriptomic data analysis of Micro Array data from human plaque samples

The MaasHPS transcriptomic data were analyzed using the R Bioconductor lumi (15) package (v2.38.0). First, we performed a variance stabilizing transformation, which is incorporated in the *lumi* package (15). Then, the robust spline normalization (RSN) algorithm in the *lumi* package was applied to normalize the data. Gene differential expression analysis was performed using the *Limma* (16) R package (v3.42.2) with Benjamini-Hochberg correction. Multiple transcript isoforms were mapped to the same gene based on the HUGO Gene Nomenclature Committee (HGNC) symbols by selecting the highest expressed transcript.

### 2.3 Plaque composition analysis

For this cohort, we measured the plaque, media, cap, necrotic core, hemorrhage, and luminal thrombus area by morphometric analysis of H&E sections. Histological features were assessed by immunohistochemistry as described by in Jin et al., 2021 (14, 17).

### 2.4 Human blood cells isolation and culture

Buffy coats were collected from healthy volunteers from Uniklinik RWTH Aachen, Germany, according to local regulations and peripheral blood mononuclear cells (PBMCs) were isolated using ficoll-paque gradient (Sigma). Subsequently, CD14^+^ monocytes were positively selected using CD14 MicroBeads (Miltenyi) according to the manufacturer’s protocol. Monocytes were pooled from 6-8 donors and plated in 96 well plates (BD#353219, black optical imaging plates) at a density of 75,000 cells/well in RPMI1640 (Thermofisher) supplemented with 10% FCS and 1% PenStrep (Gibco) and cultured in a controlled environment (37°C, 5% CO_2_). Monocytes were differentiated to macrophages using 100 ng/ml recombinant human macrophage colony-stimulating factor (rh-MCSF, Immunotools) or 5 ng/ml recombinant human granulocyte-macrophages colony-stimulating factor (rh-GMCSF, Immunotools) for 7 days.

### 2.5 Macrophage transfection

Three different transfection agents have been tested (Lipofectamin 2000 by Thermo Fisher, HiPerFect by Qiagen and Viromer Blue by Lipcalyx). Viromer Blue (Lipocalyx) gave the highest transfection efficiency and the inflammatory reponse by macrophages and was used for the transfection of primary human macrophages following the manufacturer’s protocol. The final concentration of gene-specific siRNA (Eurogentec; **Supplemental Table 1**) was 12.5 nM. Macrophages were transfected for 24h with subsequent medium change and an additional 24h resting period, after which the knockdown efficiency and the functional profile of macrophages was determined.

### 2.6 Functional profiling of macrophages by the MacroScreen

Macrophage functions were assessed by the “MacroScreen” high-content analysis (HCA) platform (18) using fluorescence-based functional assays imaged with the automated fluorescent microscope BD Pathway 855 (BD Bioscience). Nine pictures were taken per well with a 10x Olympus 0.40 NA objective and analyzed using Attovision image analysis software (BD bioscience), unless stated otherwise. A digital segmentation mask was created for each individual cell (region of interest, ROI). Morphological results (area, shape, granularity, and actin stress) were further processed using the Cell Profiler software ref 18 (19). All other data were processed by DIVA software (BD Bioscience). All plates (BD#353219, black optical imaging plates) contained eight control wells with unstimulated macrophages. Normalization to these plate controls was applied when plate to plate fluorescent intensity variation was observed. All the microscale assays were benchmarked against mesoscale counterparts. In addition, technical controls were used to validate the functional assays. Three independent experiments were performed with 6-8 replicates per condition per assay, with similar outcome. Data of a single representative experiment are shown. All experiments were performed at 37°C and 5% CO_2_ unless stated otherwise.

#### 2.6.1 Apoptosis

To analyze the percentage of apoptosis, cell nuclei were stained with Hoechst 33342 (Sigma) in complete medium for 10 minutes. Subsequently, macrophages were incubated with 1200 nM staurosporin (Sigma) for 1h. After washing with annexin binding buffer (10 mM HEPES, 140 mM NaCl and 5 mM CaCl_2_; pH of 7.4), cells were incubated with 2.5 ng/ml Annexin-V conjugated to Oregon Green 488 (Thermofisher) for 15 minutes (20). After washing with annexin binding buffer, the plate was imaged immediately. Cells incubated with culture medium only (negative control) or with staurosporine but without Annexin (positive control) served as technical controls. Cells were imaged in fresh annexin binding buffer.

#### 2.6.2 Morphology

To analyze cell morphology (i.e. size, shape, actin stress and granularity), cells were first fixed with 2% paraformaldehyde (PFA) for 15 minutes, and subsequently stained for 30 minutes with Hoechst 33342 (Sigma) and Phalloidin 594 (Santa Cruz) at room temperature. After washing with PBS, the plate was imaged using a 40x objective. To analyze the images, we used Cell Profiler software (v3.1.9, open source (21)) to measure cell area (hypertrophy), form factor (morphology), actin stress (phalloidin-Texas Red staining intensity and pattern) and granularity.

#### 2.6.3 Lipid uptake

To analyze lipid uptake, fully-differentiated macrophages were incubated for 2.5h with a mix of 8 µg/ml oxidized human low-Density Lipoprotein (oxLDL, prepared as described before (22)) and 2 µg/ml Topfluor (Avanti Polar Lipids) in complete RPMI medium immediately after preparation. Subsequently, cell nuclei were stained with Hoechst 33342 (Sigma) in complete medium and imaged in PBS thereafter. The negative control wells were incubated with Hoechst 33342 only.

#### 2.6.4 Mitochondrial stress

To analyze mitochondrial stress, macrophages were incubated with 1.200 nM staurosporin (Sigma) for 2h. Afterwards, cells were stained with 250 nM Mitotracker Red (Invivogen) and Hoechst 33342 (Sigma) for 30 minutes. Stained cells were imaged using a 20x objective. Cells incubated in absence of staurosporin served as a technical control.

#### 2.6.5 Phagocytosis (bead uptake)

To analyze phagocytosis, stimulated macrophages were incubated for 1h with 12.5 ng/ml pHrodo-labelled Zymosan beads (Thermo Fisher Scientific) per well in complete medium. Subsequently, cell nuclei were stained with Hoechst 33342 (Sigma) in complete medium and after washing with PBS, the plate was imaged. Wells (n=6-8 per plate) incubated with 25 µM cytochalasin D (Sigma) for 30 minutes prior to bead incubation, to inhibit bead uptake, served as technical control.

### 2.7 RNA isolation for RNA-seq

For RNA-Sequencing of silenced of STAT5A and STAT5B in macrophages, total RNA was isolated from macrophages using the Micro RNeasy kit (Qiagen) according to the <100,000 cells protocol. RNA quality and integrity were assessed using a TapeStation System (Agilent). Each group consisted of 4 replicates. The average RIN value per group was 8.3 or higher. The three samples with the highest RIN value per group were selected for sequencing.

### 2.8 RNA-seq library generation and sequencing

The NEBNext® Poly (A) mRNA Magnetic Isolation Module and Ultra II Directional RNA Library Prep Kit for Illumina (NEB) was used for the construction of sequencing libraries, which were then sequenced with the Illumina NextSeq 2000 System and NextSeq P3 sequencing reagent kit in a single read-mode with a read-length of 72 bp and dual unique index sequences.

### 2.9 RNA-seq data preprocessing

Sequenced reads were trimmed for adaptor sequence using cutadaptor (v3.5). Trimmed reads were mapped to the Ensembl reference human transcriptome GRCh38.p13 cDNA using kallisto (v0.46.2) and were subsequently quantified as read counts and transcript per million (TPM) at the gene level by R package tximport (v1.20.0) based on the Ensemble GRCh38 v104 GTF annotation file. Lowly expressed genes with an average read count below 5 were removed, resulting in 14,406 genes for downstream analyses.

### 2.10 Bioinformatic analyses of RNA-seq data

DESeq2 (v1.32.0) was used for gene differential expression analysis with the setting of independent filtering = TRUE and alpha = 0.05. Benjamini-Hochberg adjusted p-value was set as 0.05, together with absolute log2 fold change greater than 0 for significantly differentially expressed genes (DEGs). Using varianceStabilizingTransformation function provided in the DESeq2 package, gene expression values were transformed with the setting blind = TRUE. To explore the sample distribution, we next performed principal component analysis for all three groups, for siSTAT5A vs siCtr and for siSTST5B vs siCtr, using DESeq2 plotPCA function. This analysis was complemented with sample-wise similarity analysis, estimated by Spearman’s correlation. Gene set overrepresentation analysis (GSOA) was performed using R package clusterProfiler (v4.0.5) based on the Gene Ontology database with Benjamini-Hochberg adjusted p-value set as 0.05 for significance.

### 2.11 Phosphorylated STAT5 detection

The phosphorylation of STAT5 was measured by the Phospho-STAT5 (Tyr694) Homogeneous Time Resolved Fluorescence (HTRF) cellular kit (Cisbio) according to the manufacturer protocol using the Flexstation 3 Multimode plate reader (Molecular Devices).

### 2.12 Multiplex ELISAs

After 24h exposure of macrophages to standard lipopolysaccharide (LPS 50 ng/ml, Invivogen), supernatants were collected. V-plex custom human cytokine ELISA (Mesoscale, MSD) was performed and levels of soluble IL-10, IL-12p70, TNFα, IL-6, IL-8 and IL-1β were measured according to the manufacturer’s protocol. After 24h exposure of *ex vivo* cultured plaque tissue to the STAT5 inhibitor STAT5-in-1 (Calbiochem) and STAT5A-specific inhibitor POM-CHF-Stafia-1 (**Supplemental Figure 1**), a cell-permeable and phosphatase-stable prodrug based on the STAT5A-specific inhibitor Stafia-1 (compound 27 from the original publication (23)), plaque-conditioned supernatant was collected. V-plex custom human cytokine ELISA (Mesoscale, MSD) was performed and levels of soluble IL-10, IL-12p70, IL-6, IL-8 and IL-1β were measured according to the manufacturer’s protocol. The IL-6 levels were too high to measure. Measured cytokine concentration was adjusted for plaque slice weight.

### 2.13 TNFα ELISA

After 24h exposure of *ex vivo* cultured plaque tissue to standard lipopolysaccharide (LPS 50 ng/ml, Invivogen), plaque-conditioned supernatant was collected. The TNFα ELISA (#DY210 R&D Systems) was performed following the manufacturer’s protocol and read at 450 nm with iMark microplate absorbance reader (Bio-Rad). Measured cytokine concentration was adjusted for plaque slice weight.

### 2.14 Immunohistochemistry

Plaque tissue was fixed in 4% buffered formaldehyde solution for 24 hours, followed by a 4-hour decalcification step, subsequently embedded in paraffin, and cut into 4 µm thick sections. Deparaffinization and rehydration was performed using xylene and graded ethanol. Endogenous peroxidase activity was blocked with exposure to 0.3% H_2_O_2_ for 15 minutes. Heat induce antigen retrieval was done by heating in a water bath for 10 minutes at 97°C in a low pH target retrieval solution (K8005, DAKO, Agilent Technologies). Sections were incubated with monoclonal antibody rabbit anti human 6hosphor-STAT5 (Tyr694) (C71E5) (1:100, #9314S Cell Signaling) together with monoclonal mouse anti human CD68 (1:800, M0814 DAKO), overnight at 4°C. After washing in TBS, sections were incubated for 1h at room temperature in BrightVision poly HRP anti rabbit IgG (ImmunoLogic, VWR KDPVR55HRP) together with BrightVision poly Alkaline phosphatase anti mouse IgG (Immunologic, VWR KDPVM55AP). After washing in TBS, CD68 staining was developed using Vector Red substrate kit (SK-5100 Vector Laboratories) for 10 minutes and pSTAT5 with PolyDetector HRP green kit (Bio SB) for 5 minutes. Sections were counterstained with Mayer’s Haematoxylin (VWR International B.V., Amsterdam, The Netherlands), dehydrated, mounted and cover slipped. Sections were digitized and analyzed using the Nuance Multispectral Imaging system 3.0.2 (PerkinElmer, Inc.).

### 2.15 Plaque single-nucleus ATAC-seq data analysis

PlaqView (23) (www.plaqview.com), an online interactive web application for plaque scRNA-seq and sn-ATACSeq dataset exploration, was also used to inspect plaque cell populations for expression and chromatin accessibility of the STAT5A and STAT5B isoforms. Specifically, we interrogated the sn-ATACSeq dataset (28,316 nuclei; ~320,000 accessible sites) on the cohort of coronary artery plaques from 41 patients generated by Turner at el (24).

### 2.16 Ex vivo plaque culture

Atherosclerotic plaque samples obtained during carotid endarterectomy from 4-10 symptomatic patients (**Supplemental Table 2**) were collected in the Maastricht Pathology Tissue Collection (MPTC) in line with the Dutch Code for Proper Secondary Use of Human Tissue (http://www.fmwv.nl) and the local Medical Ethical Committee (protocol number 16-4-181). This study conforms to the Declaration of Helsinki. The endarterectomy specimens were cut into 2 mm thick sections. Subsequently, the sections were cultured in RPMI1640 (Thermofisher) supplemented with 0.1% FCS and 1% PenStrep (Gibco) and cultured in a controlled environment (37°C, 5% CO_2_) for 24 hours in presence of the general STAT5 inhibitor STAT5-in-1 (300 µM, Selleck Chemicals) or the STAT5A-specific inhibitor POM-CHF-Stafia-1 (50 µM, **Supplemental Figure 1**) (25). Control sections and inhibitor treated sections were alternating. Control sections were treated with DMSO concentration equal to the inhibitors. A multiplex ELISA was performed on the plaque-conditioned medium as described above.

### 2.17 Statistics

Data are expressed as mean ± SD, unless stated otherwise. All statistical analyses were performed using GraphPad Prism 8 software. Statistically significant differences were evaluated using the unpaired t-test for functional experiments unless stated otherwise. *p<0.05, **p<0.01, ***p<0.001 and ****p<0.0001.

## 3 Results

### 3.1 STAT5A and STAT5B are inversely correlated in human atherosclerosis

**Figure 1:**
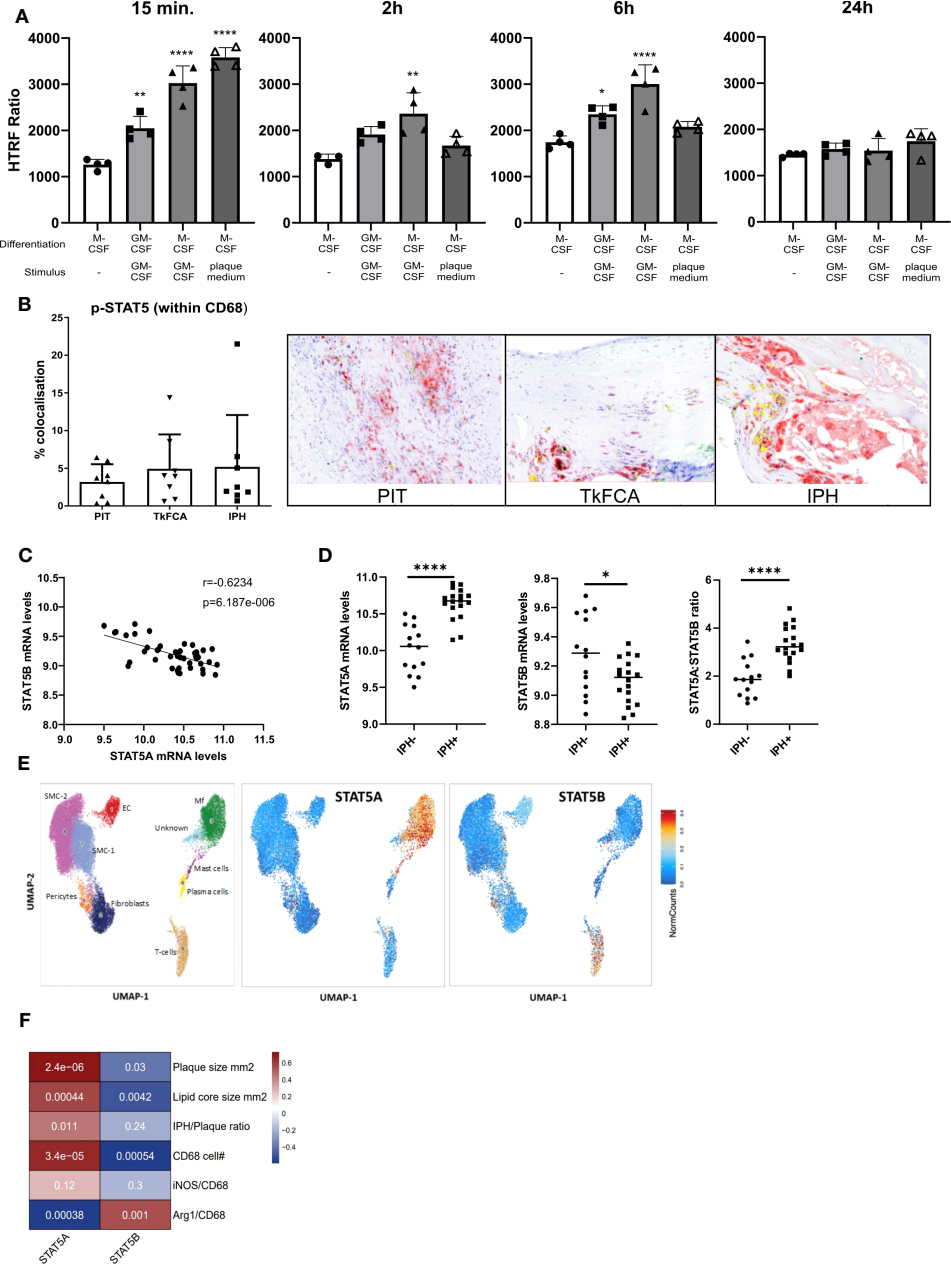
STAT5A and STAT5B in atherosclerosis. **(A)** Activation time course of STAT5 in human macrophages differentiated with GM-CSF or M-CSF and stimulated with GM-CSF (5 ng/ml) or plaque-conditioned medium (1:5 dilution with fresh medium) for indicated times. HTRF ratio was calculated as the ratio between Tyr694 phosphorylated STAT5 over total STAT5. Each dot represents a biological replicate. **(B)** IHC of CD68 (red), pSTAT5 (green) and CD68/pSTAT5 (yellow) double staining of atherosclerotic lesions with pathological intima thickening (PIT), thick fibrous cap (TkFCA) and intra-plaque hemorrhage (IPH). Representative pictures of IHC staining. **(C)** Pearson correlation of mRNA expression levels of STAT5A and STAT5B in human atherosclerotic plaques. **(D)** Log2 transformed mRNA expression levels and STAT5A:STAT5B expression ratio in intraplaque-hemorrhage-negative (IPH-) and -positive (IPH^+^) plaques. Statistically significance was assessed by unpaired t-test with Welch’s correction. **(E)** Chromosomal accessibility of STAT5A and STAT5B gene in single cells of carotid artery plaque (ATAC-Seq). Left panel indicates the annotation of clustered cell populations (EC: endothelial cells, Fibroblasts, Mf: macrophages, Mast cells; Pericytes, Plasma cells SMC: smooth muscle cells; T-cells. Color gradient indicates normalized cell counts. **(F)** Correlation of STAT5A and STAT5B expression to characteristics of human atherosclerotic plaque. Colors indicate strength of correlation. Square annotation represents p-value. Values are Mean ± SD. *p<0.05, **p<0.01, ***p<0,001, ****p<0,0001.

STAT5 inhibition was recently shown to exert anti-inflammatory effects in mouse atherosclerosis (4). In this study we specifically aimed to examine the regulation and potential role of STAT5 and its isoforms A and B in human macrophages and atherosclerosis. In line with previous literature, we found GM-CSF to induce STAT5 activation, while M-CSF did not (26). STAT5 activation by GM-CSF in M-CSF differentiated macrophages could be observed from 15 min onwards and lasted for at least 6 hours (**Figure 1A**). Prior GM-CSF-induced differentiation of macrophages with subsequent treatment with GM-CSF also led to STAT5 activation, even after seven days of differentiation (**Figure 1A**). However, M-CSF differentiated macrophages stimulated with GM-CSF exhibited a stronger activation of STAT5. Interestingly, short incubation with plaque-conditioned medium induced strong but short-lived phosphorylation of STAT5 in human macrophages (**Figure 1A**). Accordingly, STAT5 was found to be expressed and activated in human atherosclerotic plaque macrophages. Immunohistochemistry confirmed the presence of activated STAT5 in CD68^+^ plaque macrophages. Approximately 5% of all CD68^+^ cells were pSTAT5^+^ (**Figure 1B**).

Next, we examined the expression of STAT5A and STAT5B isoforms in human atherosclerosis. Surprisingly, STAT5A and STAT5B mRNA expression in human carotid artery plaque was inversely correlated (**Figure 1C**). STAT5A expression was increased, whereas that of STAT5B was reduced in hemorrhaged (IPH^+^) compared to non-hemorrhaged (IPH^-^) atherosclerotic lesions (**Figure 1D**). Of note, the ratio of STAT5A over STAT5B expression in advanced stable plaque showed an even more pronounced shift, with potential repercussions on the formation of STAT5 homo- or heterodimer in plaque (**Figure 1D**). Interestingly, analysis of overall expression of both isoforms and of genes with highest co-expression with STAT5A and STAT5B in the immune cell database of the Correlation AnalyzeR (27) revealed a highly significant positive, not negative, correlation between the A and B isoform and their co-expressed genes (**Supplemental Figure 2A**). This suggests that the inverse correlation of the A and B isoform in plaque could reflect differences in activation status of macrophages or cellular composition. Indeed, analysis of the single nucleus ATAC-Seq dataset (24) by Plaqview revealed almost exclusive chromosomal accessibility of the STAT5A gene in plaque macrophages and of STAT5B in plaque T-cells (and pericytes) (**Figure 1E**). This was in line with STAT5A and STAT5B expression profiles in the single cell RNA-Seq dataset of Wirka et al., 2019 (28) (data not shown). The significant correlation of STAT5A with plaque size (mm^2^), lipid core size and CD68^+^ macrophage content confirmed this notion (**Figure 1F**). Interestingly, Arg-1^+^/CD68^+^ cells, representing an anti-inflammatory macrophage subset, were seen to be positively correlated with STAT5B and inversely correlated with STAT5A, suggesting an association of STAT5A with macrophage phenotype and thus with transition from advanced stable to hemorrhaged plaque.

### 3.2 Expression of STAT5 in macrophages

**Figure 2:**
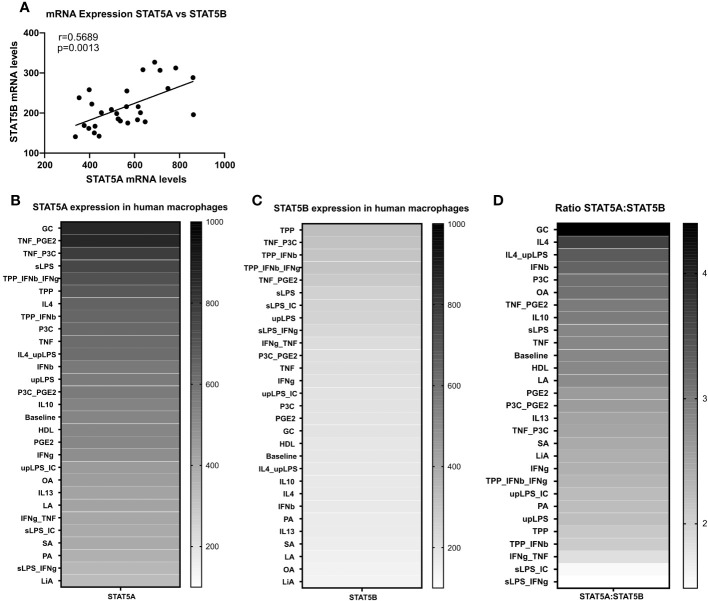
STAT5A and STAT5B expression in human macrophages. **(A)** Pearson correlation of Log2 transformed mRNA expression levels of STAT5A and STAT5B in GM-CSF differentiated human macrophages stimulated reported stimuli for 24h from Xue et al. (29). **(B, C)** mRNA expression levels of STAT5A and STAT5B in GM-CSF differentiated macrophages unpolarized (baseline) or treated with indicated stimulus obtained from Xue et al. (29). **(D)** mRNA expression ratio of STAT5A:STAT5B in unpolarized (baseline) or treated with indicated stimulus.

We next studied the expression regulation of both isoforms by a panel of 28 different atherosclerosis relevant pro- and anti-inflammatory stimuli in GM-CSF differentiated macrophages in the dataset of Xue et al. (29). Similar to the strong co-regulation of STAT5A and STAT5B in the immune cells at baseline, STAT5A and STAT5B expression was also co-regulated in activated macrophages (**Figure 2A**). In keeping with our findings in human plaque, macrophage STAT5A expression was higher than that of STAT5B in GM-CSF differentiated macrophages at baseline and after various stimulations (**Figures 2B–D**). No specific pattern could be observed for the expression of STAT5A or STAT5B in stimulated cells. However, when looking at the ratio between STAT5A and STAT5B expression levels, macrophages stimulated with anti-inflammatory stimuli such as IL-4 or IL-10 showed an increased expression of STAT5A compared to STAT5B. In contrast, cells stimulated with pro-inflammatory stimuli like tumor necrosis factor (TNF)α + interferon (IFN)γ or Lipopolysaccharide + IFNγ displayed an increased expression of STAT5B compared to STAT5A (**Figure 2D**). As immunohistochemistry analysis indicated that pSTAT5 staining was particularly prominent in subendothelial macrophages and early foam cells (**Figure 1B**; **Supplemental Figure 2B**), we investigated the expression of STAT5A and STAT5B in early foam cells. Interestingly, macrophages incubated with oxLDL for 24h showed sharply reduced expression of STAT5A and STAT5B as compared to non-foamy control macrophages (**Supplemental Figure 3**).

### 3.3 Transcriptional effects of STAT5A silencing in human macrophages

**Figure 3:**
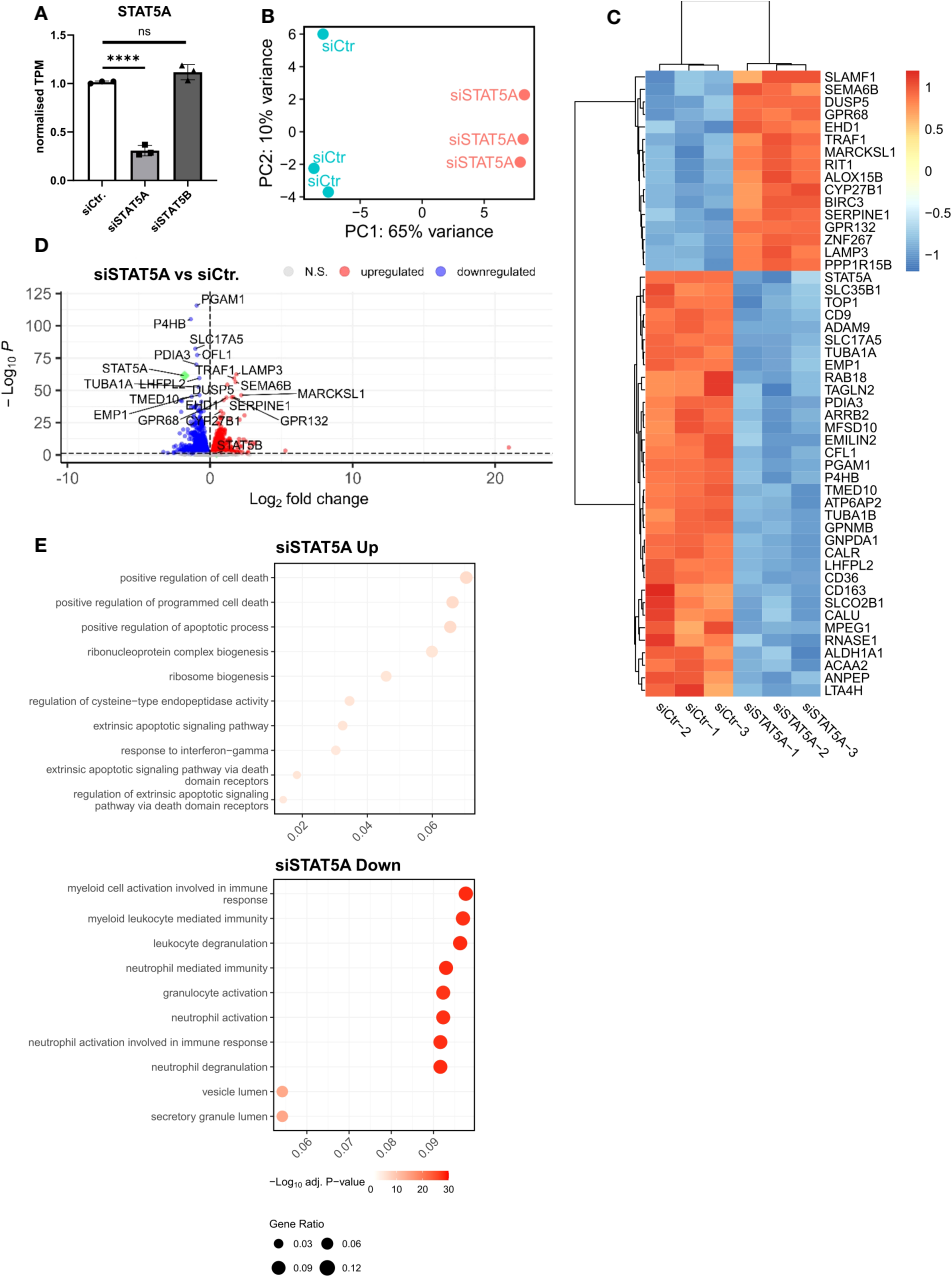
RNA-Sequencing analysis of STAT5A-silenced human macrophages. **(A)** Normalized TPM counts of STAT5A in GM-CSF differentiated human macrophages. **(B)** PCA analysis of siCtr. and siSTAT5A samples. **(C)** Heatmap representing Top50 DEGs in STAT5A-silenced human macrophages. **(D)** Volcano plot highlighting up-and downregulated genes in human macrophages after STAT5A silencing vs. control. **(E)** GSOA analysis of Top10 enriched pathways of macrophages after STAT5A silencing for down- (blue) and up- (red) regulated genes. Values are Mean± SD. *p<0.05, **p<0.01, ***p<0,001, ****p<0,0001, NS, Non-significant.

Given the much higher expression of STAT5A in atherosclerotic plaque and macrophages and the almost exclusive chromosomal accessibility of STAT5A but not STAT5B gene in plaque macrophages, we mainly focused on STAT5A. Therefore, we next investigated the transcriptional effects of STAT5A silencing in macrophages differentiated by GM-CSF, as this yielded sustained STAT5 activation. Silencing was effective with a knockdown of >70% for STAT5A. We did not observe a significant compensatory upregulation of the counter-isoform (**Figure 3A**). PCA analysis indicated strong transcriptional changes for siSTAT5A as judged from the major shift along PC1 (**Figure 3B**). This could be confirmed by Spearman’s correlation heatmap (**Supplemental Figure 4A**). A knockdown led to 1,447 up- and 1,528 downregulated differentially expressed genes (DEGs) for STAT5A silencing (**Figures 3C, D**). Enrichment analysis using CHIP-X database (Harmonizome from Maáyan Laboratories) showed a significant enrichment of known STAT5 target genes among siSTAT5 up- (p-value=0.0002) as well as down-regulated (p-value=0.00002) genes (30). GO analysis of siSTAT5A revealed over-representation of pathways associated with cell death and apoptosis for upregulated genes and with myeloid cell activation and degranulation for down-regulated genes (**Figure 3E**). The latter was confirmed by GSEA, showing significant enrichment of TNFα and IFNγ signaling genes among the STAT5A co-expressed gene set (**Supplemental Figures 4B, C**). Taken together, the transcriptional changes identified an important role of STAT5A in the immune response of human macrophages and cell survival. Of note, silencing of STAT5B, resulting in a low knockdown efficiency of 50%, only revealed 204 up- and 232 downregulated genes (**Supplemental Figure 5**).

### 3.4 STAT5A silencing impacts macrophage functions and cytokine secretion

**Figure 4:**
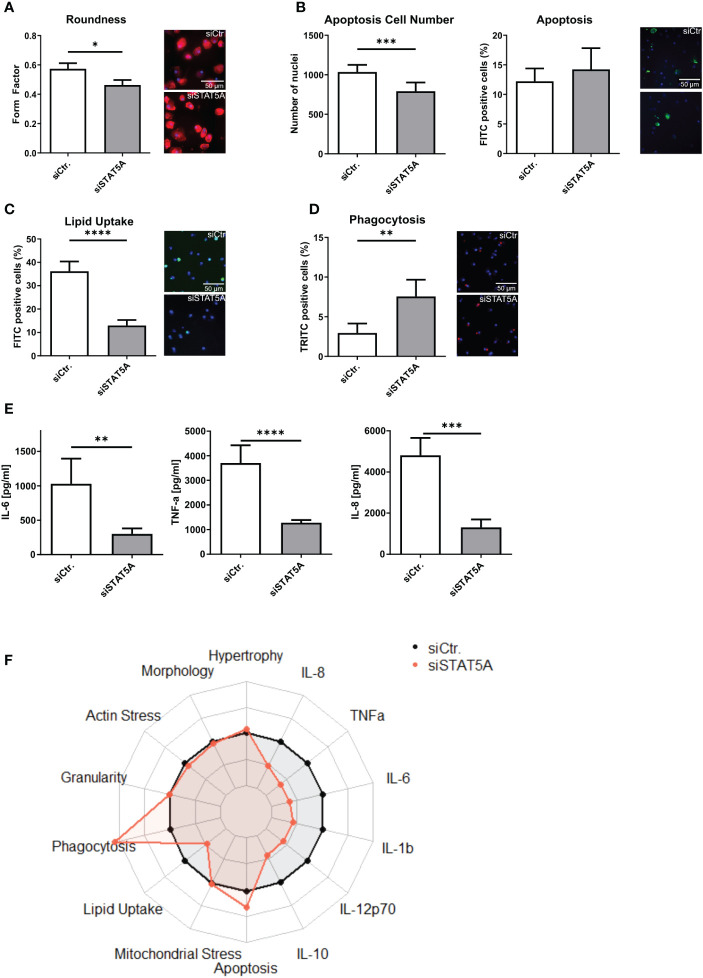
Functional effects of STAT5A on human macrophages. Functional changes were assessed for **(A)** form factor **(B)** apoptosis and cell numbers after staurosporin stimulation, **(C)** Lipid Uptake and **(D)** phagocytosis in human macrophages after silencing of STAT5A including representative pictures of significant changes (scale bar of 50µm). **(E)** Secretion of IL-6, TNFα and IL-8 after 6h LPS stimulation (50 ng/ml) in human macrophages after silencing of STAT5A. **(F)** Radarplot of macrophage functions after STAT5A silencing normalized to siCtr. samples. Three independent experiments with n=6-8 (functional assays) or n=3 (cytokine secretion). Values are Mean ± SD. *p<0.05, **p<0.01, ***p<0,001, ****p<0,0001.

The transcriptional changes of siSTAT5A in macrophages were next validated at functional level *in vitro*. While STAT5A silencing did not alter actin stress, granularity, hypertrophy and mitochondrial stress of macrophages (**Supplemental Figures 6A–D**), we observed changes in macrophage morphology, i.e. cells were more elongated after STAT5A silencing (**Figure 4A**). While upregulated genes in STAT5A silenced macrophages were enriched in apoptosis gene terms, a knockdown did reduce cell number (viability) but not the degree of apoptosis after staurosporin treatment (**Figure 4B**). In addition, cell numbers were also decreased for siSTAT5A without any additional stimulus in the phagocytosis assay (**Supplemental Figure 6E**) and after stimulation with oxLDL (**Supplemental Figure 6F**) confirming the role of STAT5A in cell viability. The most prominent functional effects of STAT5 knockdown involved phagocytosis and lipid uptake. Silencing STAT5A sharply reduced oxLDL accumulation capacity (**Figure 4C**; **Supplemental Figure 6G**). Interestingly, this was accompanied by increased phagocytosis ability (**Figures 4D, F**). These changes prompted us to investigate the expression of key receptors of these processes. Interestingly, STAT5A silencing led to significant suppression of Peroxisome proliferator-activated receptor (PPAR)γ and cluster of differentiation (CD)36 and concomitant upregulation of ATP-binding cassette transporter (ABC)G1 and scavenger receptor A1 (SRA1), potentially underlying the cholesterol metabolism and phagocytosis changes (**Supplemental Figure 6H**). On the other hand, and in keeping with the enrichment of immune terms in siSTAT5A downregulated genes, IL-6 and TNFα and IL-8 release was significantly reduced in siSTAT5A macrophages (**Figures 4E, F**). The secretion of IL-10, IL-12p70 and IL-1β did not show significant differences, albeit those values were close to the detection threshold (**Supplemental Figure 6I**). It is worth noting that despite the low degree of STAT5B silencing, it did reduce the secretion of the pro-inflammatory cytokines TNFα and IFNγ without affecting additional functional characteristics of macrophages (**Supplemental Figure 7**).

**Figure 5:**
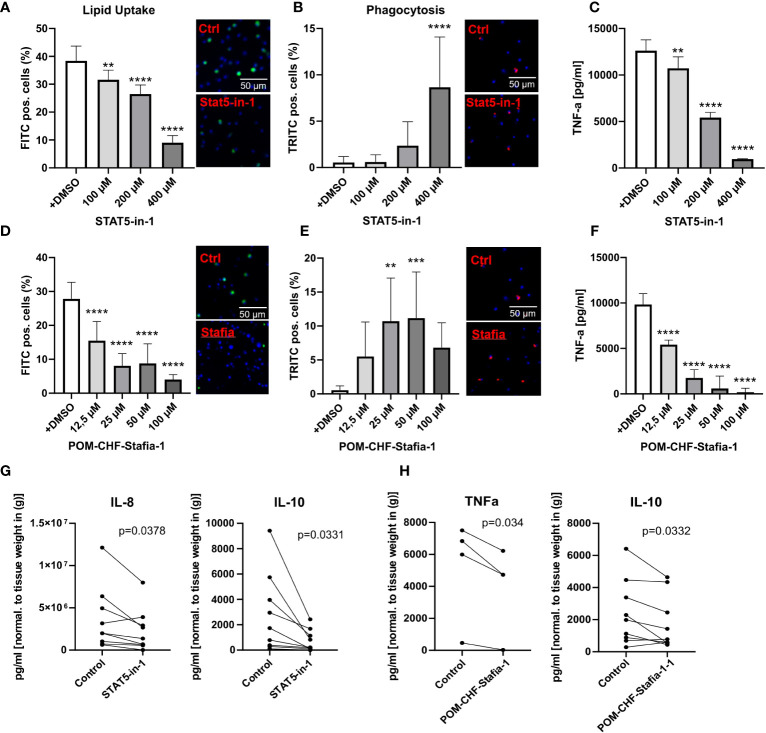
Functional effects of STAT5 inhibitors on human macrophages and human plaque tissue. **(A, D)** Lipid Uptake assay of human macrophages treated with the inhibitors STAT5-in-1 **(A)** and POM-CHF-Stafia-1 **(D)** 2h prior to assay. **(B, E)** Phagocytosis assay of human macrophages treated with the inhibitors STAT5-in-1 **(B)** and POM-CHF-Stafia-1 **(E)** 2h prior to assay. Pictures indicated significant changes (scale bar of 50µm). **(C, F)** TNFα secretion of human macrophages treated with the inhibitors STAT5-in-1 **(C)** and POM-CHF-Stafia-1 **(F)** 2h prior LPS stimulation for 6h. **(G, H)** Cytokine secretion of ex vivo plaque tissue treated with the inhibitors **(G)** STAT5-in-1 for IL-8 and IL-10 and **(H)** POM-CHF-Stafia-1 for TNFα and IL-10. Paired dots represent a single patient. Statistically significance was assessed by paired t-test. For functional analysis, 8 replicates per group were used. Values are Mean± SD. *p<0.05, **p<0.01, ***p<0,001, ****p<0,0001.

Importantly, these siRNA knockdown findings could be largely recapitulated with STAT5 inhibitors, STAT5-in-1, a non-selective cell-permeable inhibitor which acts by binding to its SH2 domain (31), and POM-CHF-Stafia-1, a cell-permeable and phosphatase stable prodrug of the STAT5-specific inhibitor Stafia-1 (25)). Both STAT5-in-1 and POM-CHF-Stafia-1 induced a dose-dependent decrease in lipid uptake (**Figures 5A, D**) and increase in phagocytosis (**Figures 5B, E**) while they also dose-dependently decreased TNFα secretion after LPS stimulation (**Figures 5C, F**). Of note, STAT5-in-1 was demonstrated to reduce pSTAT5 content of macrophages by approx. 85% (**Supplemental Figure 8A**), confirming the reported inhibitory activity (31).

### 3.5 STAT5 inhibition impairs macrophage pro-inflammatory functions in human atherosclerotic plaques *ex vivo*

*In vitro* data of STAT5A knockdowns indicate a prominent role of STAT5A in key macrophage functions. As a measure of the *in vivo* relevance of our findings, we studied effects of STAT5 and STAT5A inhibition on human plaque tissue sections *ex vivo*. After processing, plaque sections were cultured for 24h with the inhibitor at the indicated concentration. As seen *in vitro*, STAT5 inhibition markedly suppressed IL-8 secretion. Furthermore, STAT5 inhibition significantly reduced the expression of IL-10 whereas IL-12, IL-1β and TNFα was not changed (**Figure 5G**; **Supplemental Figure 8B**). Likewise, POM-CHF-Stafia-1 quenched TNFα and IL-10 secretion in human atherosclerotic plaque (**Figure 5H**), while secretion of IL-1β, IL-8 and IL-12 was unchanged (**Supplemental Figure 8C**). Within this multiplex ELISA, IL-6 levels were too high to draw conclusions. Due to potential different pharmacokinetics between cells *in vitro* and tissue *ex vivo* as well as a different cell composition in plaques, these data did not completely confirm the previous *in vitro* results. However, the overall effect of reduced secretion of cytokines, in particular pro-inflammatory cytokines, could be observed *ex vivo* as well.

## 4 Discussion

Prior work on STAT5 has reported a reduction of atherosclerotic plaques in mice after STAT5 inhibition (4). However, this and other research on STAT5, not only in macrophages, did not focus on the role of the isoforms STAT5A and STAT5B (4, 32). In this study, we were able to show an inverse correlation between STAT5A and STAT5B in atherosclerotic plaques with STAT5A being strongly associated with plaque macrophages. Knockdown of STAT5A induced transcriptional changes altering the expression of genes associated with apoptosis, phagocytosis, cholesterol metabolism and the immune response. Silencing of STAT5A not only dampened the secretion of IL-6, IL-8 and TNFα, it also reduced lipid uptake, yet increased phagocytosis and apoptosis in human macrophages, mostly confirming the observed transcriptional changes. These data are to our knowledge the first to shed light on the regulatory role of STAT5A and their functional implications in human macrophages.

We demonstrated that silencing and pharmacological inhibition of STAT5 in macrophages strongly reduced the uptake of oxLDL, which is in line with previous reports in murine macrophages and atherosclerosis (4). In pulmonary alveolar macrophages, STAT5 knockout was seen to lead to alveolar proteinosis and altered lipid metabolism, accompanied by a reduced expression of PPARγ (32). We identified STAT5A, the isoform showing highest expression in macrophages *in vitro* and in plaque (single cell ATAC-Seq), to be involved in these effects in human macrophages, as STAT5A silencing strongly reduced lipid uptake and PPARγ expression. Conversely, macrophages’ ability to phagocytose increased upon knockdown of STAT5A. The increase in phagocytosis in macrophages after silencing STAT5A could be explained by an increase of SRA1 expression in these cells. As to the augmented lipid uptake upon STAT5A silencing, it is interesting to note that the expression several key receptors in lipid handling were changed (PPAR and CD36 down-, ABCA1 upregulated), suggesting a causal role in this effect. Considering the role of lipid uptake and phagocytosis in atherogenesis, these functional changes induced by STAT5A silencing might strongly impact the initiation and transition from stable to unstable plaques in atherosclerosis. Importantly, the knockdown of STAT5A was induced after differentiation, hence not affecting this process. STAT5A DEGs were also enriched in cell death regulation genes, which was confirmed at functional level. Whether or not this has repercussions for the survival of GM-CSF differentiated macrophages under the prevailing pro-oxidant and proinflammatory conditions in atherosclerotic plaque is still unknown and needs further investigation.

A limitation of the study is the low knockdown efficiency of the STAT5B silencing (~50%). Considering this and the fact that STAT5A is the predominant isoform in plaque macrophages, our experiments do not allow to draw firm conclusions on the existence and relevance of functional differences of STAT5A and STAT5B silencing. STAT5B seemed to dampen cytokine secretion. The lack of further functional effects of STAT5B silencing on macrophages, could well be due to the limited 50% knockdown. Further study in macrophages with more pronounced STAT5B deficiency will be need to shed light on functional overlap and differences between these two STAT5 isoforms in macrophages.

The observed functional changes for STAT5A on lipid uptake and its reported impact on cell death (33) are in line with the significant correlation of this isoform with CD68^+^ cell content and lipid core size of human plaque. Moreover, the negative correlation with anti-inflammatory macrophages in plaque is in agreement with the observed anti-inflammatory effects of STAT5A knockdown. Interestingly, the B-isoform displayed an inverse correlation with these plaque traits, but this probably reflects the reciprocal regulation of both isoforms in plaque. Of note, in human plaque, STAT5B seems to be mainly expressed by T-cells, hinting to an indirect role of T-cell STAT5B on these plaque traits. In contrast to its role in apoptosis and lipid uptake, STAT5A hampered the expression of pro-inflammatory cytokines such as TNFα, IL-6 and IL-8 after LPS stimulation. STAT5A has been shown to interact with NF-κB (34) to specifically induce the expression of IL-6 (35). These observations are consistent with our observed changes in pro-inflammatory cytokine secretion in macrophages.

In human atherosclerotic plaques, we could confirm the presence of activated STAT5. Moreover, we observed the colocalization of pSTAT5 with plaque macrophages. This is in line with previous work showing increased phosphorylation of STAT5 in atherosclerosis in mice (4). GM-CSF has been identified as one of the significant activators of STAT5 (36) which levels increase in atherosclerotic lesions during the transition from stable to unstable plaque (37, 38). Interestingly, plaque-conditioned medium exhibited an even stronger yet much shorter activation of STAT5 than GM-CSF alone. However, we were not able to distinguish between phosphorylated STAT5A and STAT5B. Nevertheless, our results suggest that STAT5A is the dominant isoform in macrophages (**Figures 1**, **2**) and important for their functions (**Figure 4**). Note that in this study, we only looked at the expression of isoforms but not the activation status. The strong activation of STAT5 by plaque-conditioned medium, however, suggests the possibility of additional factors that could affect the expression and the activation of STAT5 in macrophages. Considering the wide variety of stimuli and factors in atherosclerotic plaques that affect the phenotype of macrophages, a more elaborate analysis of STAT5A in polarized macrophages would be of interest including evaluation of isoform-specific activators. Furthermore, due to a reduction of STAT5A expression in foam cells, in this study we did not focus on the effects of STAT5A in foam cells. Recent single cell studies revealed that in human plaque only a minor portion of plaque macrophages are bona fide foam cells (39, 40). Additionally, according to the studies conducted by Pan et al. (2020) and Alsaigh et al. (2020) on PlaqueView, it has been demonstrated that GM-CSF is primarily produced by T cells and NK cells. In human atherosclerosis, T cells are predominantly localized in the shoulder subendothelial regions, which primarily non-foamy macrophages reside (41), suggesting that GM-CSF stimulation of foamy macrophages is physiologically less likely to occur.

To further substantiate the observed functional changes in human macrophages in relation to human atherosclerosis, we treated human plaque tissue *ex vivo* with a general STAT5 inhibitor and a STAT5A specific inhibitor. Wang et al. reported smaller atherosclerotic plaques after STAT5 inhibition in mice (4). In our human plaque tissue slice experiments, we observed decreased the secretion of TNFα, IL-8 and IL-10 after STAT5 inhibition, suggestive of a pro-inflammatory activity of STAT5 in plaque and identifying macrophage STAT5 as a candidate for intervention in human atherosclerosis as well. Thus, STAT5 and specifically STAT5A could serve as an attractive therapeutic target for atherosclerosis.

In conclusion, STAT5A strongly affects the phenotype of human macrophages. Reduced activation resulted in a less inflammatory type of macrophages, which could clarify previous observations of reduced atherosclerosis upon STAT5 inhibition in mice.

## Data availability statement

The datasets presented in this study can be found in online repositories. The names of the repository/repositories and accession number(s) can be found below: GSE163154 and GSE217893 (GEO).

## Ethics statement

The studies involving humans were approved by Maastricht Pathology Tissue Collection (MPTC) in line with the Dutch Code for Proper Secondary Use of Human Tissue (http://www.fmwv.nl) and the local Medical Ethical Committee (protocol number 16-4-181). The studies were conducted in accordance with the local legislation and institutional requirements. The human samples used in this study were acquired from by-product of an carotid endarterectomy operation. Written informed consent for participation was not required from the participants or the participants’ legal guardians/next of kin in accordance with the national legislation and institutional requirements.

## Author contributions

JN conceived the study, processed the data, conceived, performed and analyzed the biological experiments, and wrote the manuscript with input from all co-authors. HJ performed the computational analyses. AR performed biological experiments. BM provided human plaque tissue. CS contributed to the methodology by providing knowledge of and access to the Multiplex system. DM-K synthezised the STAT5A inhibitor. LT, PG, MD and TB offered critical revision. EB conceived and supervised the study, provided funding and critical feedback at all stages. All authors contributed to the article and approved the submitted version.

## Supplementary material

Supplementary Figure 1 Structure of the prodrug POM-CHF-Stafia-1 against STAT5A.

Supplementary Figure 2 **(A)** Expression correlation of STAT5A and STAT5B, and their co-expressed genes. Correlation AnalyzeR was used to assess the correlation between STAT5A and STAT5B, and their co-expressed genes in the general immune database. **(B)** Immunohistological images stained for CD68 (red) and pSTAT5 (green) and CD68/pSTAT5 (yellow) in IPH plaques (1 and 2), plaques with a thickened fibrous cap (3) and pathological intima thickening (4).

Supplementary Figure 3 Gene expression of STAT5A and STAT5B in human macrophages (THP1) stimulated with (oxLDL) or without (Control) oxLDL (25mg/ml) for 24h. Changes in gene expression were measured by RT-PCR. Ubiquitin was used a house keeping gene. Values are Mean±SD.

Supplementary Figure 4 **(A)** Spearman’s Correlation heatmap of RNA-Sequencing data for siCtr. and siSTAT5A. **(B, C)** Gene set enrichment analysis of STAT5A co-expressed genes for **(B)** IFNg signalling and **(C)** TNFA signalling.

Supplementary Figure 5 **(A)** TPM of STAT5B normalized to siCtr. **(B)** PCA analysis of siCtr. and siSTAT5B. **(C)** Heatmap representing Top50 DEGs in STAT5B-silenced macrophages. **(D)** Volcano plot highlighting up-and downregulated genes in macrophages with STAT5B-silencing vs. control. **(E)** GO analysis of macrophages after STAT5B silencing for down- and up-regulated genes.

Supplementary Figure 6 Functional changes in **(A)** Granularity, **(B)** Actin stress, **(C)** Mitochondrial stress, **(D)** Hypertrophy, **(E)** Number of nuclei in Phagocytosis assay, **(F)** Number of nuclei in lipid uptake assay, and **(G)** Mean fluorescent intensity of positive cells in lipid uptake in human macrophages after silencing of STAT5A and STAT5B. Three independent experiments with n=5-8. Mean±SEM. **(H)** Absolute mRNA levels of PPARG, ABCG1, CD36 and SRA1 in siCtr. siSTAT5A and siSTAT5B macrophages. **(I)** Secretion of IL-12p70, IL-1β, and IL-10 after 6h LPS stimulation (50 ng/ml) in human macrophages after silencing of STAT5A. Three independent experiments with n=3. Values are Mean±SD. *p<0.05, **p<0.01, ***p<0,001, ****p<0,0001.

Supplementary Figure 7 Functional changes in **(A)** Hypertrophy, Granularity, Actin stress, Roundness, **(B)** Mitochondrial stress, **(C)** Lipid Uptake and mean fluorescent intensity of lipid uptake, **(D)** phagocytosis assay, **(E)** Apoptosis assay, Number of nuclei after staurosporin treatment, in lipid uptake assay, in phagocytosis assay in human macrophages after silencing of STAT5B. Three independent experiments with n=5-8. Mean±SEM. **(F)** Secretion of IL-6, TNFα, IL-8, IL-12p70, IL-1β, and IL-10 after 6h LPS stimulation (50 ng/ml) in human macrophages after silencing of STAT5B. Three independent experiments with n=3. Values are Mean±SD. *p<0.05, **p<0.01, ***p<0,001, ****p<0,0001.

Supplementary Figure 8 **(A)** Phosphorylation of STAT5 after GM-CSF stimulation and treatment with STAT5 inhibitor STAT5-in-1 measured with the Phospho-STAT5 (Tyr694) Homogeneous Time Resolved Fluorescence (HTRF) cellular kit (Cisbio). **(B, C)** Cytokine secretion of ex vivo plaque tissue treated with the inhibitors (B) STAT5-in-1 for TNFα, IL-12 and IL-1β and **(C)** POM-CHF-Stafia-1 for IL-12, IL-1β and IL-8. Paired dots represent a patient. Statistically significance was assessed by paired t-test.
