# Supplementary figures and images for: GM-CSF-activated STAT5A regulates macrophage functions and inflammation in atherosclerosis
Source: Front Immunol. 2023 Oct 18;14:1165306. doi: 10.3389/fimmu.2023.1165306 (PMC10619680; doi:10.3389/fimmu.2023.1165306)

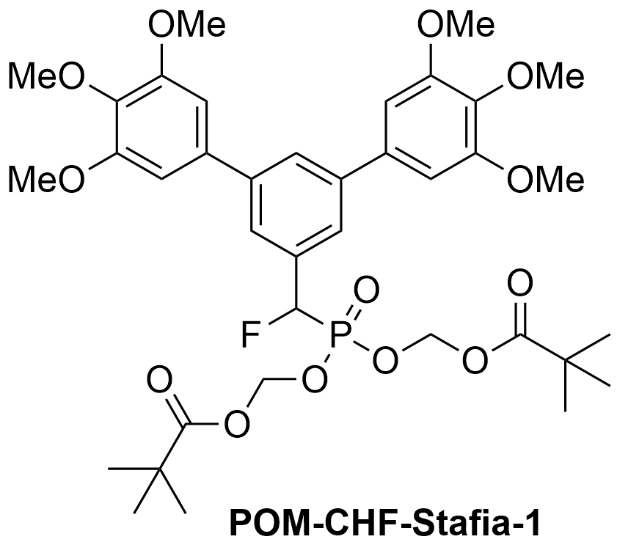

Supplement: Supplementary Figure 1 — Structure of the prodrug POM-CHF-Stafia-1 against STAT5A. [file Image_1.png]

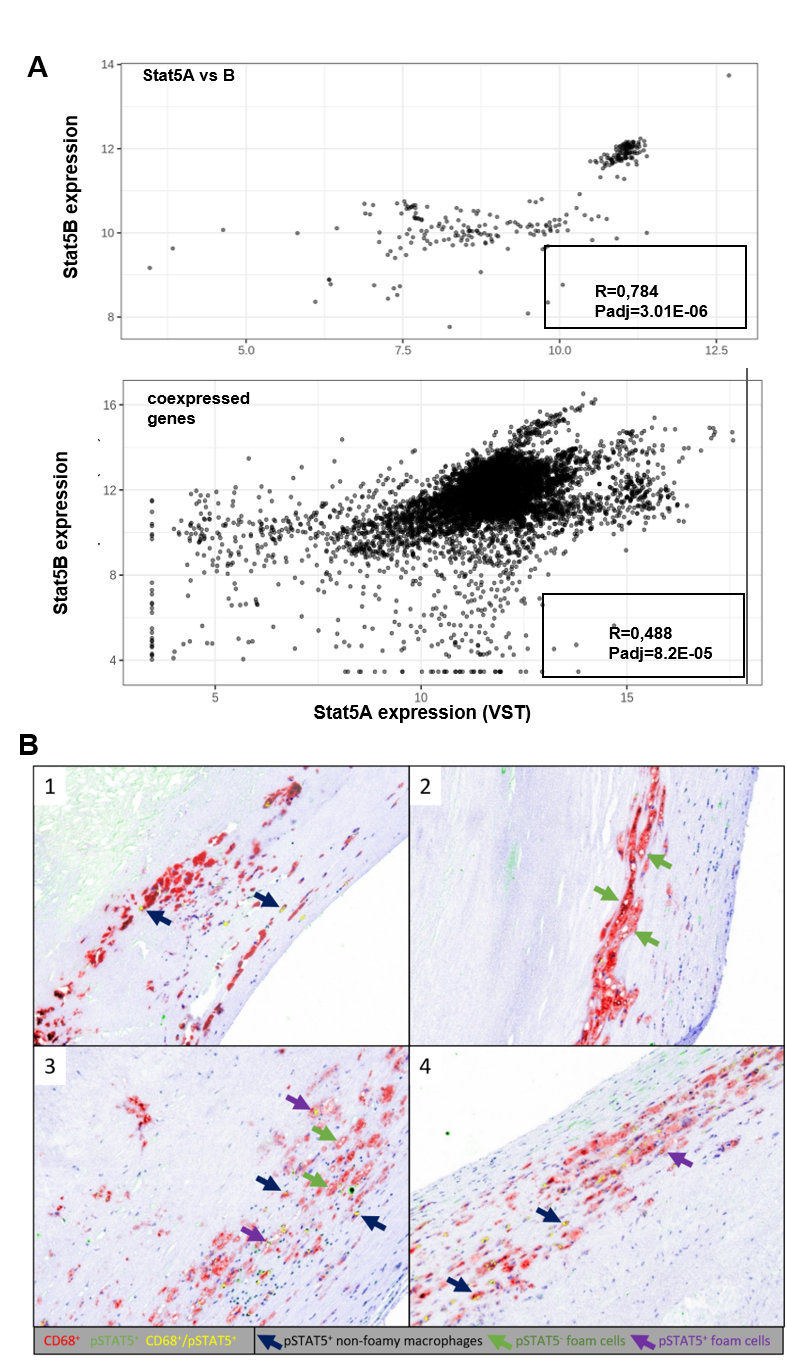

Supplement: Supplementary Figure 2 — (A) Expression correlation of STAT5A and STAT5B, and their co-expressed genes. Correlation AnalyzeR was used to assess the correlation between STAT5A and STAT5B, and their co-expressed genes in the general immune database. (B) Immunohistological images stained for CD68 (red) and pSTAT5 (green) and CD68/pSTAT5 (yellow) in IPH plaques (1 and 2), plaques with a thickened fibrous cap (3) and pathological intima thickening (4). [file Image_2.png]

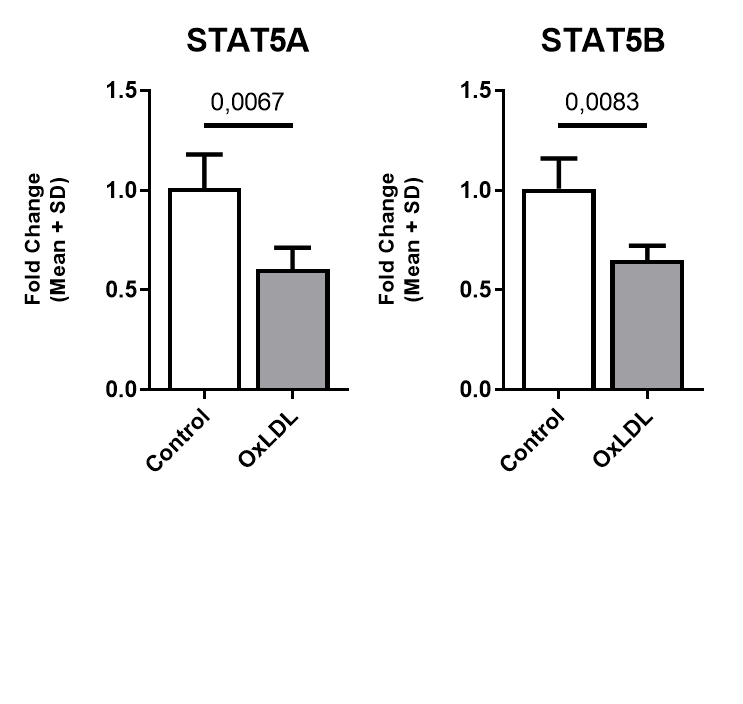

Supplement: Supplementary Figure 3 — Gene expression of STAT5A and STAT5B in human macrophages (THP1) stimulated with (oxLDL) or without (Control) oxLDL (25mg/ml) for 24h. Changes in gene expression were measured by RT-PCR. Ubiquitin was used a house keeping gene. Values are Mean±SD. [file Image_3.png]

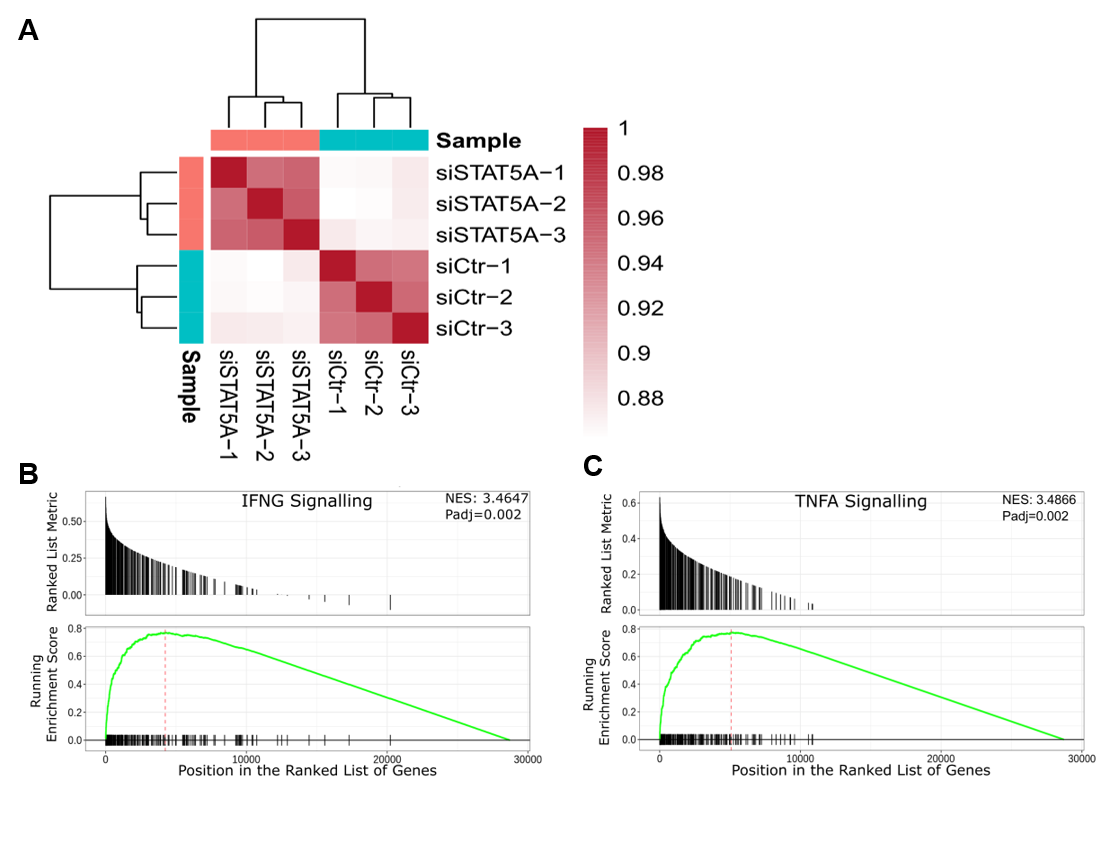

Supplement: Supplementary Figure 4 — (A) Spearman’s Correlation heatmap of RNA-Sequencing data for siCtr. and siSTAT5A. (B, C) Gene set enrichment analysis of STAT5A co-expressed genes for (B) IFNg signalling and (C) TNFA signalling. [file Image_4.png]

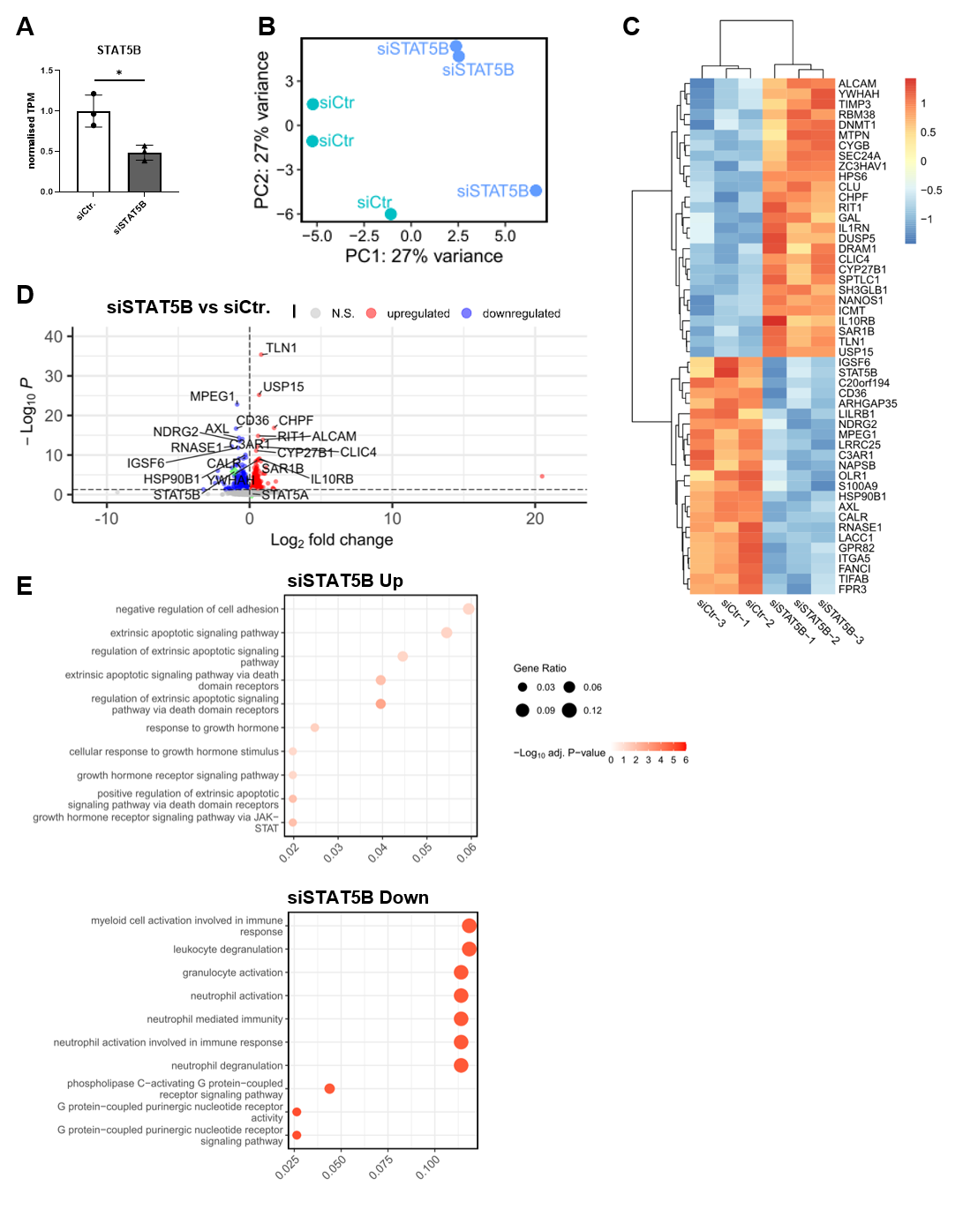

Supplement: Supplementary Figure 5 — (A) TPM of STAT5B normalized to siCtr. (B) PCA analysis of siCtr. and siSTAT5B. (C) Heatmap representing Top50 DEGs in STAT5B-silenced macrophages. (D) Volcano plot highlighting up-and downregulated genes in macrophages with STAT5B-silencing vs. control. (E) GO analysis of macrophages after STAT5B silencing for down- and up-regulated genes. [file Image_5.png]

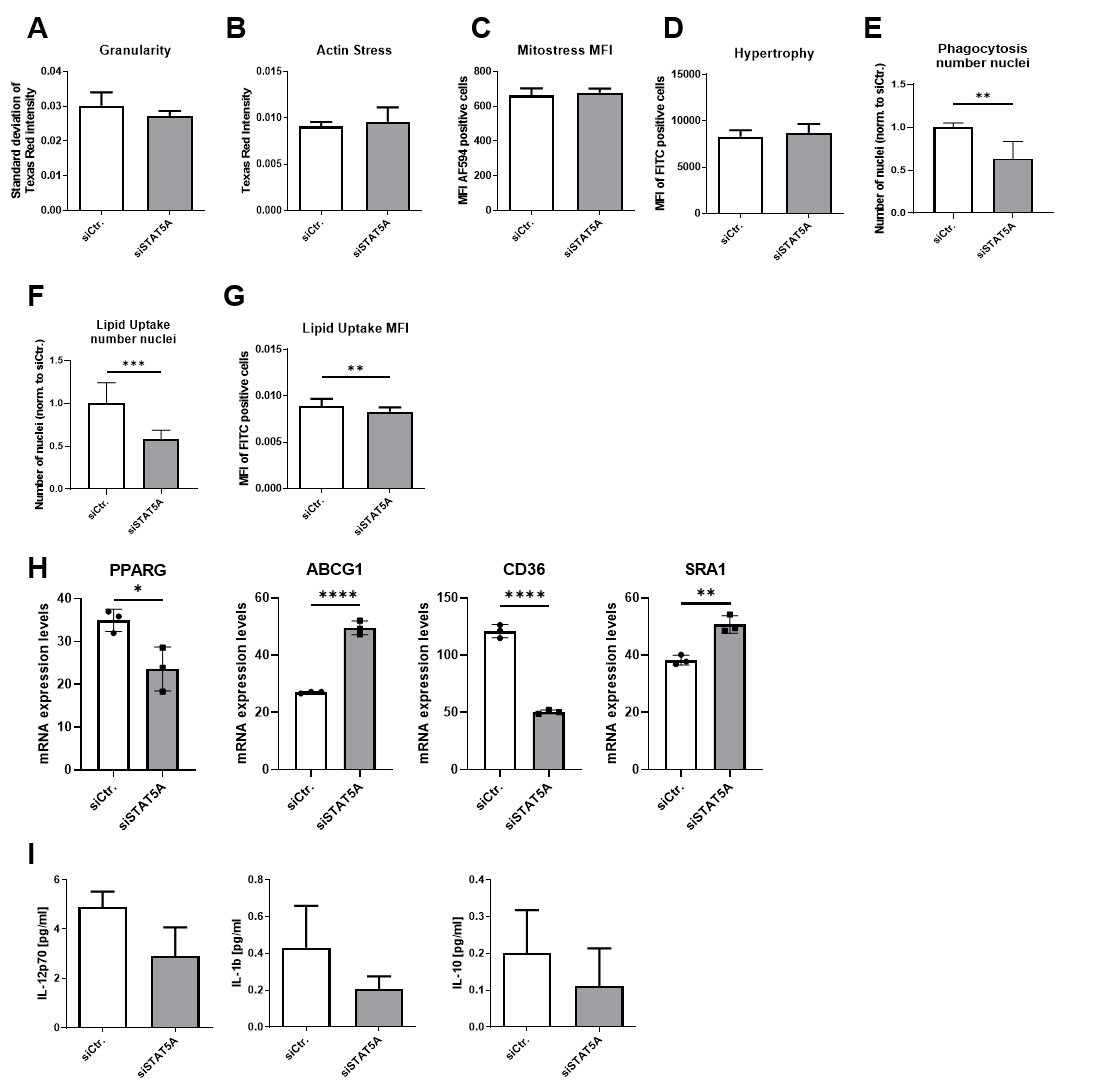

Supplement: Supplementary Figure 6 — Functional changes in (A) Granularity, (B) Actin stress, (C) Mitochondrial stress, (D) Hypertrophy, (E) Number of nuclei in Phagocytosis assay, (F) Number of nuclei in lipid uptake assay, and (G) Mean fluorescent intensity of positive cells in lipid uptake in human macrophages after silencing of STAT5A and STAT5B. Three independent experiments with n=5-8. Mean±SEM. (H) Absolute mRNA levels of PPARG, ABCG1, CD36 and SRA1 in siCtr. siSTAT5A and siSTAT5B macrophages. (I) Secretion of IL-12p70, IL-1β, and IL-10 after 6h LPS stimulation (50 ng/ml) in human macrophages after silencing of STAT5A. Three independent experiments with n=3. Values are Mean±SD. *p<0.05, **p<0.01, ***p<0,001, ****p<0,0001. [file Image_6.png]

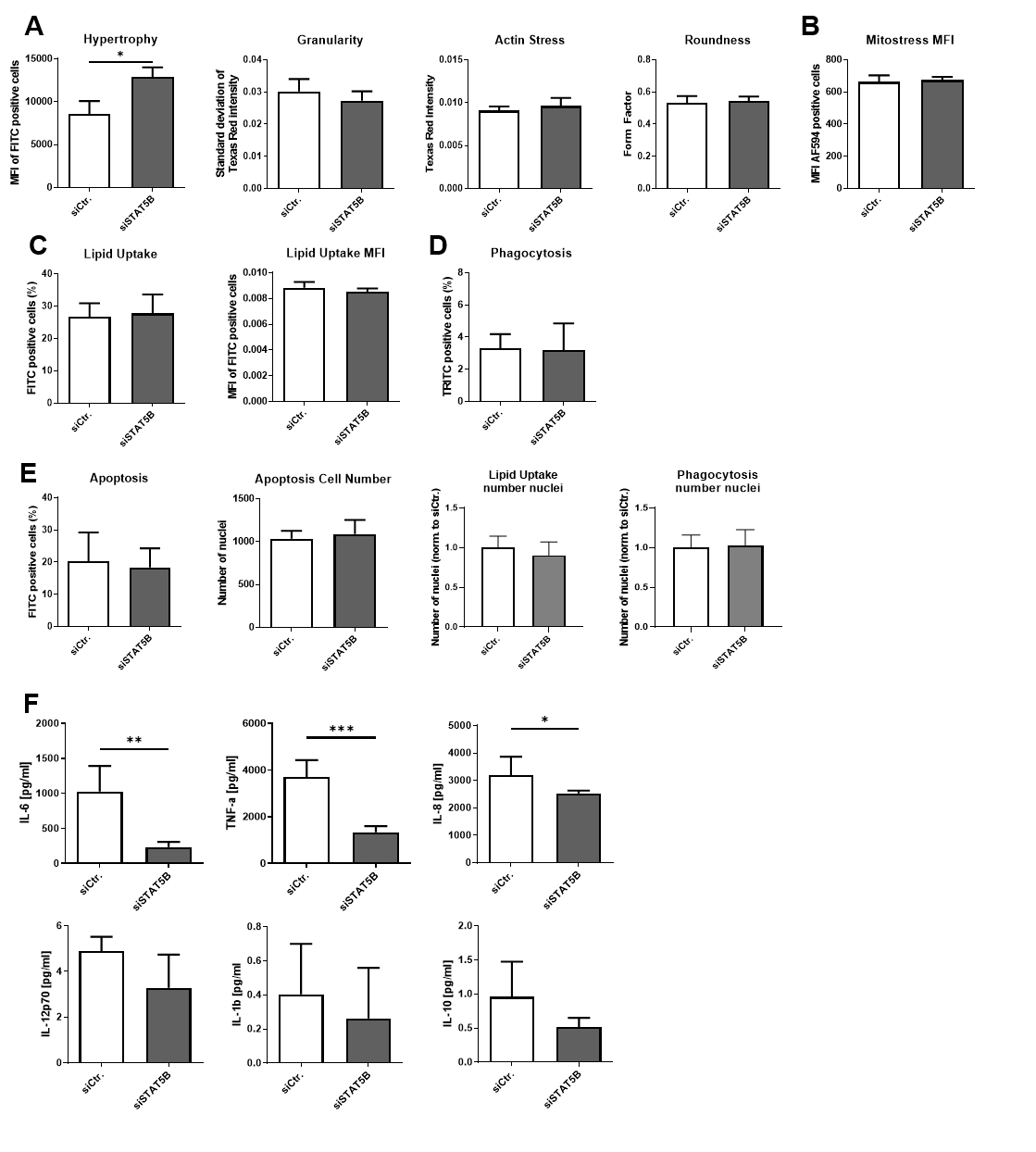

Supplement: Supplementary Figure 7 — Functional changes in (A) Hypertrophy, Granularity, Actin stress, Roundness, (B) Mitochondrial stress, (C) Lipid Uptake and mean fluorescent intensity of lipid uptake, (D) phagocytosis assay, (E) Apoptosis assay, Number of nuclei after staurosporin treatment, in lipid uptake assay, in phagocytosis assay in human macrophages after silencing of STAT5B. Three independent experiments with n=5-8. Mean±SEM. (F) Secretion of IL-6, TNFα, IL-8, IL-12p70, IL-1β, and IL-10 after 6h LPS stimulation (50 ng/ml) in human macrophages after silencing of STAT5B. Three independent experiments with n=3. Values are Mean±SD. *p<0.05, **p<0.01, ***p<0,001, ****p<0,0001. [file Image_7.png]

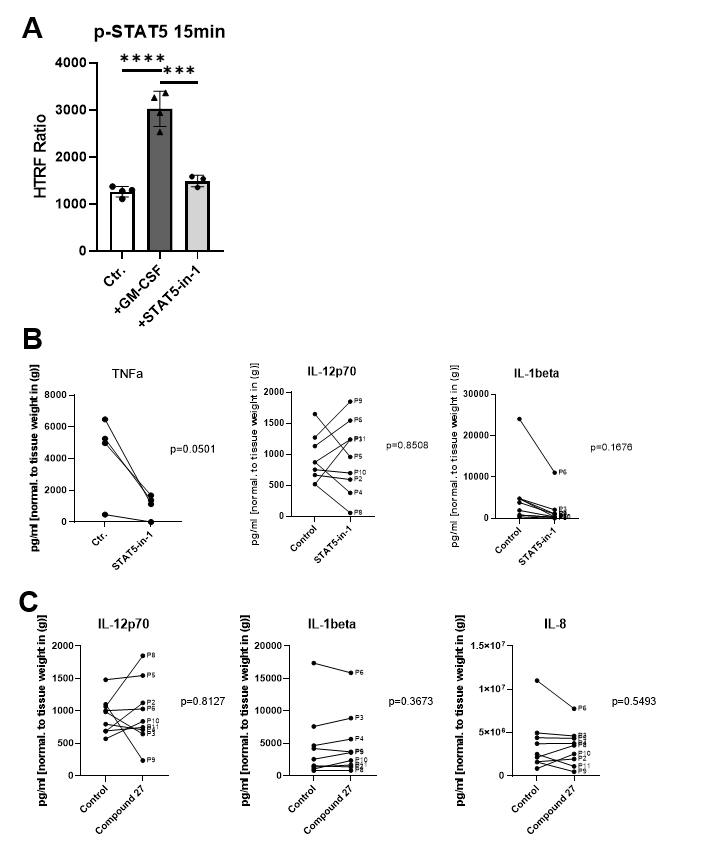

Supplement: Supplementary Figure 8 — (A) Phosphorylation of STAT5 after GM-CSF stimulation and treatment with STAT5 inhibitor STAT5-in-1 measured with the Phospho-STAT5 (Tyr694) Homogeneous Time Resolved Fluorescence (HTRF) cellular kit (Cisbio). (B, C) Cytokine secretion of ex vivo plaque tissue treated with the inhibitors (B) STAT5-in-1 for TNFα, IL-12 and IL-1β and (C) POM-CHF-Stafia-1 for IL-12, IL-1β and IL-8. Paired dots represent a patient. Statistically significance was assessed by paired t-test. [file Image_8.png]
