# Supplementary material for: GM-CSF-activated STAT5A regulates macrophage functions and inflammation in atherosclerosis
Source: Front Immunol. 2023 Oct 18;14:1165306. doi: 10.3389/fimmu.2023.1165306 (PMC10619680; doi:10.3389/fimmu.2023.1165306)
Supplement: Supplementary file 9 [file Table_1.docx]

Supplementary Material

Table 1: Sequence of used siRNAs.

| siRNA | Sequence |
| --- | --- |
| siSTAT5A | UGUCACUGAAGAGGAUCA |
| siSTAT5B | CUCAGUAGAUCUUGAUAAU |

Table 2: Year of birth and sex of patients used for *ex vivo* plaque culturing

| Plaque # | Year of birth | Sex |
| --- | --- | --- |
| #1 | 1941 | M |
| #2 | 1948 | M |
| #3 | 1958 | Not indicated |
| #4 | 1932 | M |
| #5 | 1945 | F |
| #6 | 1950 | M |
| #7 | 1940 | F |
| #8 | 1929 | F |
| #9 | 1950 | M |
| #10 | 1936 | F |
